# A new synthetic protocol for coumarin amino acid

**DOI:** 10.3762/bjoc.9.30

**Published:** 2013-02-06

**Authors:** Xinyi Xu, Xiaosong Hu, Jiangyun Wang

**Affiliations:** 1 Laboratory of Noncoding RNA, Institute of Biophysics, Chinese Academy of Sciences, Beijing 100101, China; 2 Graduate University of the Chinese Academy of Sciences, Beijing 100864, China; 3 Department of Chemistry, School of Science, Wuhan University of Technology, Wuhan 430070, China

**Keywords:** coumarin, fluorescent probe, halogen derivatives, non-natural amino acid, Pechmann condensation

## Abstract

The hydrochloride of the racemic amino acid (2-(7-hydroxycoumarin-4-yl)ethyl)glycine, which can serve as a fluorescent probe in proteins, and two halogen derivatives of it, were synthesized by using a new synthetic protocol in five steps. It is less costly and relatively easy to prepare this kind of fluorescent amino acid with the new synthetic method. Furthermore, it can be applied to synthesize other derivatives of the coumarin amino acid with some specific properties.

## Introduction

**Figure 1:**
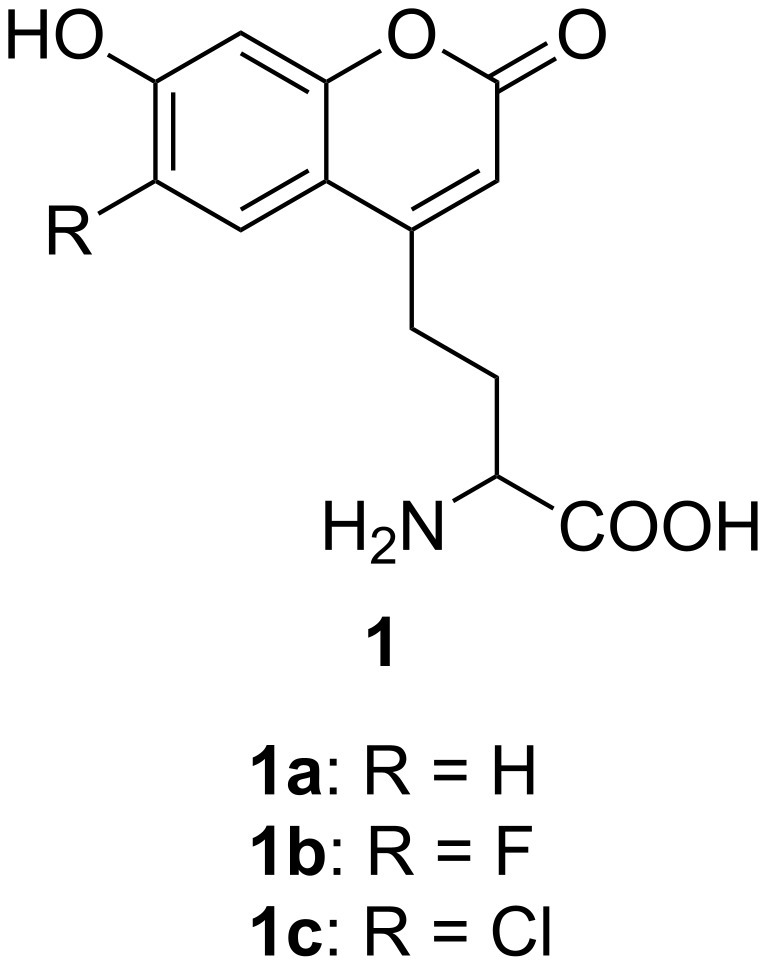
The chemical structure of a series of fluorescent amino acids. (**1a**) α-(2-(7-hydroxycoumarin-4-yl)ethyl)glycine; (**1b**) α-(2-(6-fluoro-7-hydroxycoumarin-4-yl)ethyl)glycine; (**1c**) α-(2-(6-chloro-7-hydroxycoumarin-4-yl)ethyl)glycine.

Since α-L-(2*-*(7-hydroxycoumarin-4-yl)ethyl)glycine (**1a**, Figure 1), a fluorescent non-natural amino acid, was genetically incorporated at a defined site in proteins in living organisms for the first time by Schultz and co-workers [1] there have been more and more applications related to it [2–8]. Compound **1a** is of great interest to scientists because the 7-hydroxycoumarin moiety has a high fluorescence quantum yield and a large Stokes shift. Its excellent fluorescence properties make it a great candidate to substitute green fluorescent protein (GFP) in the application of fluorescent labeling of living cells. Compared with GFP, compound **1a** is small, and can be incorporated at any defined site in proteins; whereas GFP is large, which will cause significant perturbation, and can only be fused to the C- or N-terminus of the target protein [9]. The coumarin amino acid **1a** (Figure 1) is sensitive to both pH and solvent polarity, which makes it a good probe to investigate protein functionalities and biological processes related to them. The following examples are several applications of it. Shan and co-workers used compound **1a** to form a FRET pair with the dye BODIPY-Fl to study the dynamics of protein–protein interactions [2]. Wang and co-workers genetically incorporated **1a** to a position near to amino acids, which can be phosphorylated to investigate how phosphorylation affects the fluorescence properties of **1a**, and the variation in the fluorescence intensity can be used to probe the phosphorylation status of certain amino acids [3]. Chapman and co-workers studied the FtsZ protein with genetically incorporated **1a** [4]. The fluorescence of **1a** was utilized to study the assembling of FtsZ in vivo, especially how the Z-ring is formed by FtsZ. This cannot be achieved by using GFP labeling technique since GFP is relatively insensitive to the pH and the solvent polarity of the solution. Many more examples of the usage of **1a** have also been reported [5–8]. Due to the high importance of **1a**, there has been a great need for a highly efficient and practical protocol for its synthesis.

There are two major different approaches available in the literature to synthesize compound **1a**. Approach 1 was reported by Schultz and co-workers, and it was the first synthetic protocol to provide **1a** as an enantiomerically pure amino acid in L-configuration [1]. In this approach, *N*-α-Cbz-L-glutamic acid α-benzyl ester was first converted into the side-chain β-keto ester and then it was reacted with resorcinol in methanesulfonic acid to afford **1a**. The biggest shortcoming of this approach is that the purification of the final product requires a costly preparative reversed-phase HPLC system. Other drawbacks of this approach include that the reactant Z-Glu-OBzl is expensive, and it is difficult to characterize some of the intermediates formed. Approach 2 was designed by Braun and Dittrich to provide an alternative path for the synthesis of **1a** [10]. It started from a coumarinylacetic acid, which was then reduced to an alcohol by borane-dimethyl sulfide. After the phenolic hydroxy group was protected with a *tert*-butyl(dimethyl)silyl group, the alcohol was converted into a bromide and was used to alkylate a glycine enolate synthon to afford an imine. All the protecting groups were then removed and racemic amino acid **1a** was afforded. This approach prevents the tedious and costly HPLC purification step used in approach 1. However, it can only provide compound **1a** as a racemic mixture, and some reagents used are not readily available. Due to the great importance of coumarin amino acid, we designed a new synthetic protocol for compound **1a** and two further halogen derivatives (**1b** and **1c**). This new approach avoids some of the problems discussed above. It has a good overall yield and only requires reagents that are relatively cheap or easy to prepare. Compounds **1b** and **1c** have different sensitivities to pH and solvent polarity, and can serve as new fluorescent probes in a variety of applications.

## Results and Discussion

**Scheme 1:**
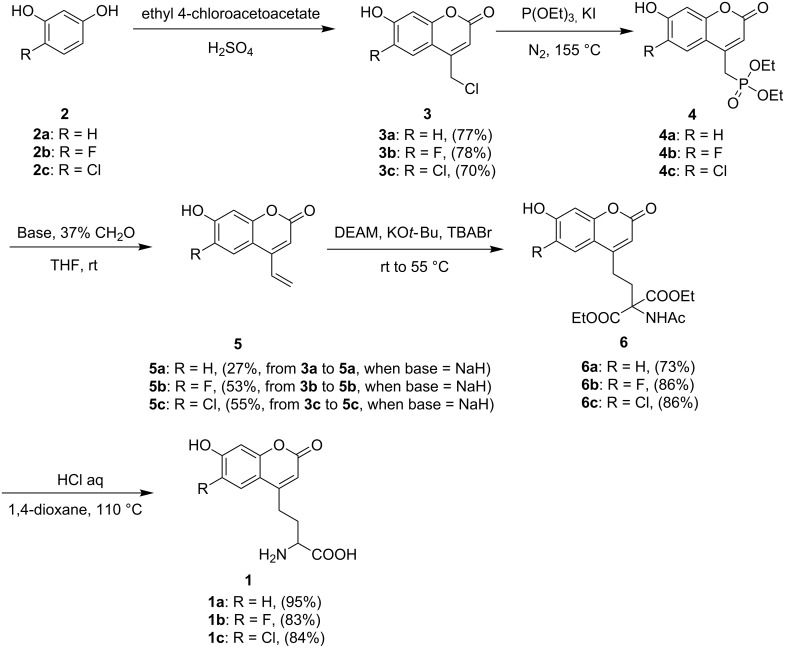
Synthetic route to fluorescent amino acids **1a**, **1b** and **1c**.

Scheme 1 gives an outline of the new protocol used to synthesize compound **1**. First, the coumarin ring with a 4-chloromethyl group (compound **3**) was formed through Pechmann condensation [11] from ethyl 4-chloroacetoacetate and resorcinol or its 6-halogenated derivatives (**3a**, 77.4% yield; **3b**, 78.0%; **3c**, 70.0%). By suspending compound **3** in triethyl phosphate with a catalytic amount of sodium iodide and heating the mixture under reflux, phosphonate **4** was prepared and used directly in the next step without purification. Compound **4** was first treated with sodium hydride or other bases and then reacted with formaldehyde to form terminal alkene **5** through a Horner–Wadsworth–Emmons reaction [12] (two-step overall yields are 27%, 53% and 55% for **3a** to **5a**, **3b** to **5b** and **3c** to **5c**, respectively, if sodium hydride was used). In the presence of potassium *tert*-butoxide and a catalytic amount of tetrabutylammonium bromide, alkene **5** reacted with diethyl acetamidomalonate (DEAM) [13] to form malonate **6** in high yield (**6a**, 73.1% yield; **6b**, 86.0%; **6c**, 86.0%). It was then heated under reflux in aqueous HCl solution (12 M) to completely remove the protecting groups to afford the racemic coumarin amino acid **1** (**1a**, 95% yield; **1b**, 83%; **1c**, 84.3%).

This is a short and practical approach for the synthesis of coumarin amino acid **1**. The Pechmann condensation was chosen to assemble the coumarin ring, since the yield is high and derivatives with substituents at the 5-, 6- or 8-position can be prepared [14–16]. In this paper, we report the synthesis of 6-fluoro and 6-chloro derivatives of compound **1a** through Pechmann condensation. The functional group introduced at these positions will further improve the fluorescent property of the coumarin amino acids, or add new chemical handles to the coumarin ring for some specific investigations. The Horner–Wadsworth–Emmons reaction was applied to install the terminal alkene at the 4-position. Compared with the Wittig reaction, the Horner–Wadsworth–Emmons reaction has a significant advantage: The resulting phosphate byproduct can be readily separated, whereas the byproduct triphenylphosphine oxide generated in the Wittig reaction is difficult to remove [17]. The effect of the base used in the Horner–Wadsworth–Emmons reaction on the reaction yield was investigated (Table 1). Three different bases including sodium hydride, 1,8-diazabicycloundec-7-ene (DBU) and potassium *tert*-butoxide (KO*t*-Bu) were used, and the reactions were carried out at 25 °C for 5 h. DBU gave the best yield in the synthesis of **5a**, **5b** or **5c**, whereas there was no reaction at all when KO*t*-Bu was used. This indicates that KO*t*-Bu is not basic enough to deprotonate the α-hydrogen of phosphonate **4** at 25 °C. However, when the reaction temperature was raised to 55 °C, the deprotonation happened and compound **5** was afforded with good yield (Table 2). This indicates that the reaction temperature has a significant effect on the Horner–Wadsworth–Emmons reaction. Since the coumarin ring could be opened under basic conditions, especially at high temperature [18], we need to choose proper base and reaction temperature to get the optimized yield of terminal alkene **5**. Based on the experimental results, DBU is the best base among the three, and the reaction can be carried out at room temperature to obtain a relatively good yield. Alkylation of DEAM is widely used to synthesize α-amino acids [13], which was also applied here to prepare the coumarin amino acids. Through Michael addition reaction of DEAM to terminal alkene **5**, malonate **6** was formed in high yield, and it was followed by the total hydrolysis of compound **6** with concentrated aqueous HCl solution to afford the coumarin amino acid **1**. We found that the hydrolysis needed a high concentration of aqueous HCl solution (12 M) and a relatively long reaction time (10 h) to reach completion.

**Table 1:** Effect of different bases on the yield of compounds **5a**, **5b** and **5c**^a^.

Compound	NaH	DBU	KO*t*-Bu

**5a**	27%	45%	No reaction
**5b**	51%	59%	No reaction
**5c**	45%	55%	No reaction

^a^Reaction temperature: 25 °C; reaction time: 5 h; solvent: THF; equiv of compound **4**/equiv of base = 1/5.

**Table 2:** Effect of temperature on the yield of compounds **5a**, **5b** and **5c**^a^.

Compound	25 °C	55 °C

**5a**	No reaction	52%
**5b**	No reaction	47%
**5c**	No reaction	48%

^a^Base: KO*t*-Bu; reaction time: 5 h; solvent: THF; equiv of compound **4**/equiv of base = 1/5.

Though this new protocol also affords the coumarin non-natural amino acids in racemic form, similar to approach 2, it has some advantages that make it practical for the preparation of these fluorescent amino acids. First, the overall yields of the products are good compared with the two approaches reported**,** and all the reagents are commercially available and relatively cheap. Second, a tedious HPLC purification step is not necessary and only flash chromatography is required. Last, but most important, derivatives with a substituent at the 6-position can be prepared, which greatly expands the usage of this synthetic protocol. Coumarin non-natural amino acids with a substituent at the 5- or 8-position can also be prepared and a respective study is ongoing in our laboratory.

**Figure 2:**
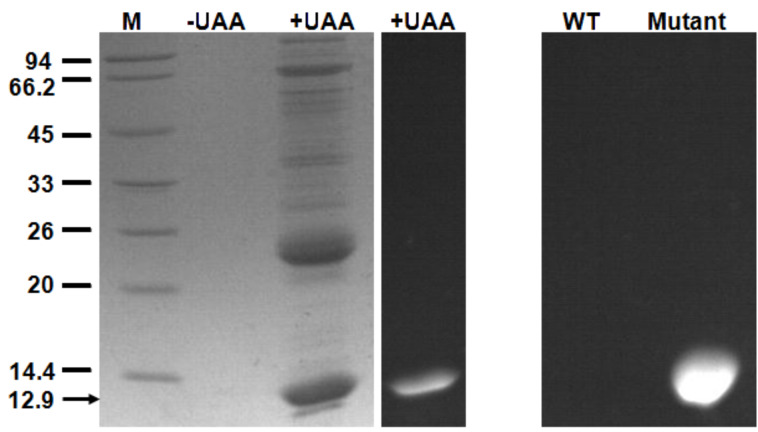
Coomassie-stained SDS-PAGE (left) of TAG38 mutant thioredoxin (indicated by the black arrow) expression in the presence and absence of 1 mM **1a**. The right panel shows the fluorescence image of wild-type and TAG38 mutant thioredoxin.

The L-enantiomer was proved to be able to be incorporated into a protein exclusively from the racemic mixture. In the presence of the synthetase CouRS and *Mj*tRNA^Tyr^_CUA_, in *E. coli* [1], an amber codon was substituted for Ile-38 in thioredoxin-1 (TRX-TAG38), and protein expression was carried out in the LB medium with the addition of 1 mM of racemic coumarin amino acid **1a**. The protein expression was also carried out without **1a** as a negative control. The synthetase/tRNA pair could uniquely specify **1a** in response to the TAG codon, which made the expression of TRX-TAG38 possible. The protein was then purified and analyzed by the SDS-PAGE gel (Figure 2). It showed that the full length protein (12.9 kDa) was only expressed in the presence of **1a**; even the final product was racemic. Since thioredoxin has no intrinsic fluorescence, the fluorescent band corresponding to mutant thioredoxin in the right panel of Figure 2 must come from incorporated **1a**. The L-enantiomer is assumed to be accepted exclusively from the racemic mixture since there is not any report indicating that D-amino acids exist in proteins. A related report proved that in the incorporation of a non-natural amino acid into a protein, the L-enantiomer is accepted exclusively from the racemic mixture [19]. Nevertheless, crystallization of the TAG38 mutant thioredoxin is under way to obtain the X-ray crystal structure of it, which will give direct proof of the exclusive incorporation of the L-enantiomer of **1a**.

**Figure 3:**
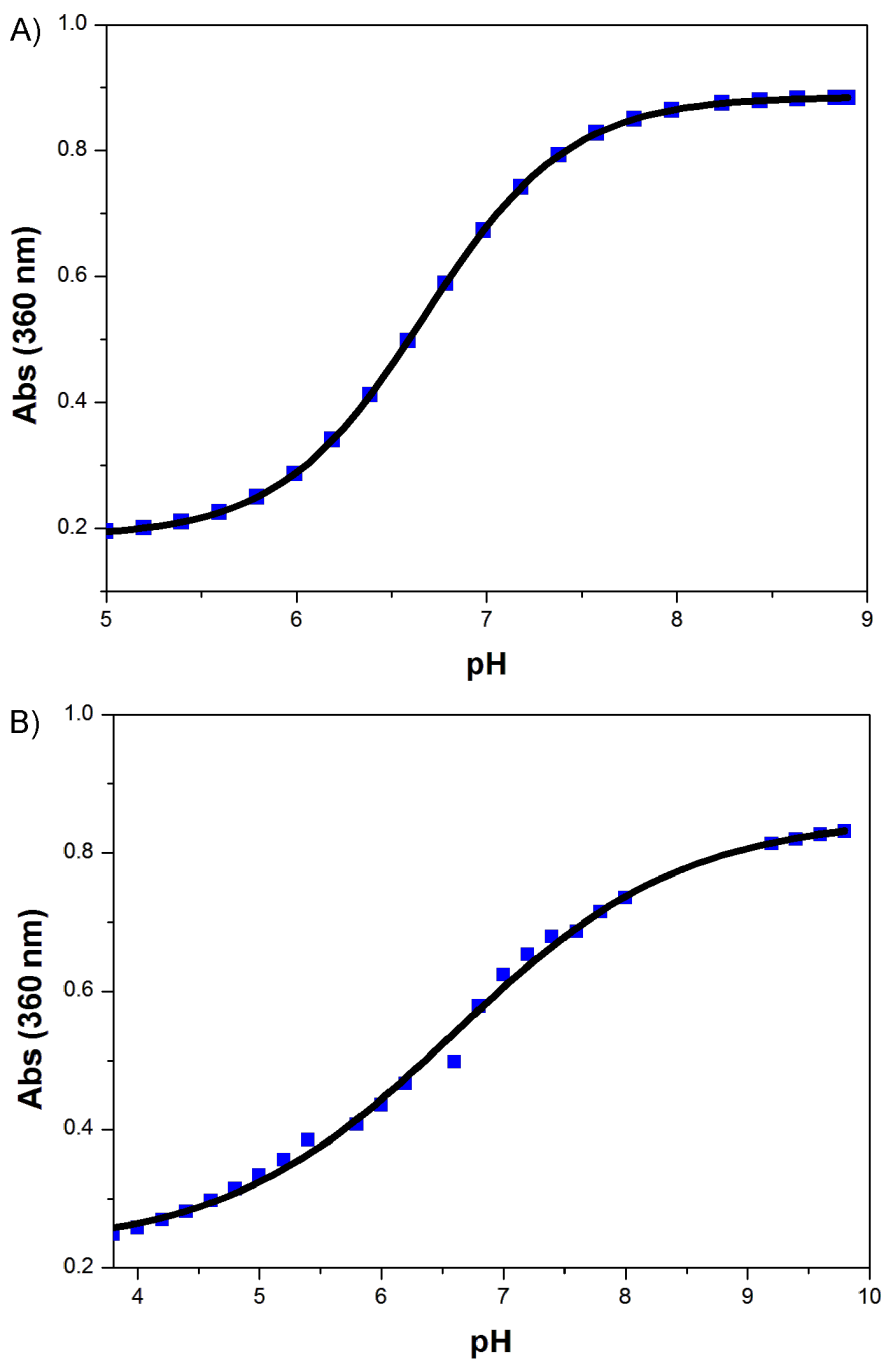
Absorbance of compounds **1b** and **1c** at 360 nm as a function of pH value. (A) Absorption spectrum of 50 μM compound **1b** in 200 mM sodium phosphate buffer (pH 5.8–8.0), 200 mM sodium acetate buffer (pH 3.7–5.6) or 50 mM Tris-HCl buffer (pH 8.2–8.9). (B) Absorption spectrum of 25 μM compound **1c** in 200 mM sodium phosphate buffer (pH 5.8–8.0) or sodium acetate buffer (pH 3.7–5.6).

**Figure 4:**
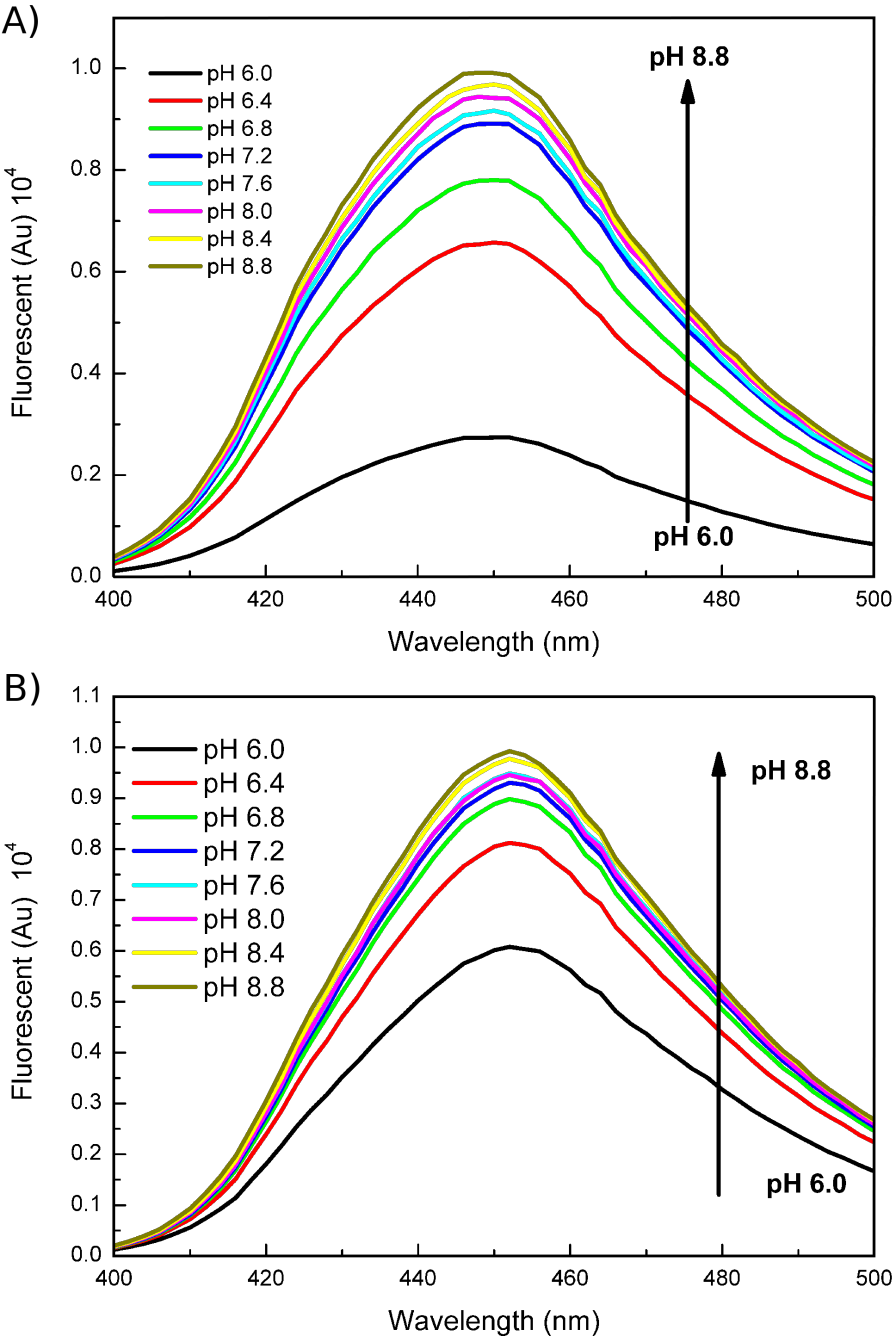
Effect of the pH value of the solution on the fluorescence emission spectra of compounds **1b** and **1c**. (A) Fluorescence emission spectrum of compound **1b**. (B) Fluorescence emission spectrum of compound **1c**.

6-Halogenated derivatives were also synthesized with this new protocol; these derivatives should have similar extinction coefficients and quantum yields as compound **1a**, according to the literature [20–21]. However, their p*K*a values are significantly different from those of **1a**, as shown in Table 3. The p*K*a values are calculated from the absorbance at 360 nm at different pH values illustrated in Figure 3, by using the Henderson–Hasselbalch equation. The halogenation of **1a** at the 6-position decreases the p*K*a value, which makes compounds **1b** and **1c** good substitutes for **1a** in fluorescent labeling and other investigations in biological systems. Screening for the synthetase/tRNA pair for **1b** and **1c** is under way. Fluorescent emission spectra of **1b** and **1c** at different pH values were both acquired (Figure 4). The fluorescence intensities of these two compounds are greatly enhanced upon increasing pH value of the solutions. This fluorescence property can be used to monitor the pH value of some acidic organelles, which may be difficult or even impossible for other investigating technologies. For the 6-fluorinated compound **1b**, its ^19^F NMR is another important property suitable for probing the biological system. Coupling the fluorescent emission spectrum with its ^19^F NMR spectrum, it will provide a very powerful means in biological investigations and analysis with small molecules.

**Table 3:** p*K*a values of compounds **1a**, **1b** and **1c** and their corresponding wavelengths of maximum emission.

Compound	p*K*a	Wavelength of maximum emission

**1a**	7.8	456 nm
**1b**	6.6	448 nm
**1c**	6.3	452 nm

## Conclusion

In summary, a new protocol for the synthesis of several fluorescent coumarin non-natural amino acids in good yields was designed. This protocol only requires relatively cheap reagents and five reaction steps in total. The separation and purification processes are much easier and an HPLC purification step is unnecessary. Thus, it is more economical and less tedious compared with previously reported protocols. 6-Halogenated coumarin non-natural amino acids have different fluorescence properties and other functionalities, which makes them good probes for various biological studies. That a series of related coumarin non-natural amino acids can be prepared with this synthetic protocol is its most important advantages. Other derivatives with a 5- and 8-substituent are being synthesized and their fluorescence properties will be studied. The drawback of this protocol is that we cannot obtain the pure L-enantiomer, and only a racemic mixture was synthesized. However we proved that the L-enantiomer can be incorporated into protein exclusively, which indicated that the racemic product is good enough for biological studies. Nevertheless, pursuit of a synthetic protocol affording the pure L-enantiomer is still our next goal.

By the chemical synthesis and genetic encoding of the fluorescent non-natural amino acids, fluorescent groups can easily be incorporated at defined sites of proteins directly in living organisms. The fluorescent group wouldn’t cause significant perturbations on proteins due to its small size, thus, it greatly extends the scope of fluorescence imaging techniques. Compound **1a** has a p*K*a of 7.8 and only its conjugate base is fluorescent. This limits its usage in the fluorescence imaging in vivo, such as in the study of receptor-mediated endocytosis. The protein involved in the endocytosis needs to be translocated from cytoplasm, which has a pH around 7, to the endosomes and lysosomes, which have pH values around 5 [22]. Since the fluorescence of compound **1a** is relatively weak at acidic conditions, compound **1b** and **1c** with lower p*K*a are important and thus synthesized. They will be more fluorescent in an acidic environment, which makes them better probes for endocytosis than compound **1a**. With the new synthetic protocol, compound **1b** and **1c** now can be prepared in a straightforward manner. The remaining unsolved problem in our work is on the molecular biology side, the focus of which is the screening of the specific aminoacyl-tRNA synthetases capable of recognizing **1b** or **1c**. Once we get the synthetases, compound **1b** and **1c** will be very useful probes of organellar pH and pH-dependent cellular processes. More coumarin amino acids with specific properties can also be prepared by using our new synthetic protocol, which makes it an important one in this research area.

## Supporting Information

File 1 Experimental and analytical data.
