# Supplementary material for: A new synthetic protocol for coumarin amino acid
Source: Beilstein J Org Chem. 2013 Feb 6;9:254–9. doi: 10.3762/bjoc.9.30 (PMC3566773; doi:10.3762/bjoc.9.30)
Supplement: File 1 — Experimental and analytical data. [file Beilstein_J_Org_Chem-09-254-s001.pdf]

Supporting Information

for

A new synthetic protocol for coumarin amino acid

Xinyi Xu[1,2], Xiaosong Hu*[1,3] and Jiangyun Wang*[1]

Address: [1]Laboratory of Noncoding RNA, Institute of Biophysics, Chinese Academy of Sciences, Beijing 100101, China, [2]Graduate University of the Chinese Academy of Sciences, Beijing 100864, China and [3]Department of Chemistry, School of Science, Wuhan University of Technology, Wuhan 430070, China

Email: Xiaosong Hu* - xhu@moon.ibp.ac.cn; Jiangyun Wang* - jwang@ibp.ac.cn

* Corresponding author

Experimental and analytical data

Experimental

General remarks: All reagents were purchased from commercial suppliers and used without further purification. Flash chromatography was carried out with silica gel (200–300 mesh). Analytical TLC was performed with silica gel GF_{254} plates, and the products were tested by UV detection. ^{1}H NMR spectra were recorded at 500 MHz (Varian DD2) and ^{13}C NMR spectra were recorded at 125 MHz (Varian DD2). Chemical shifts (δ) are reported in ppm with TMS as internal standard, and spin–spin coupling constants (*J*) are given in Hz. ^{19}F NMR spectra were recorded at 470 MHz (Varian DD2) and were reported in ppm with TFA as internal standard. HRMS (ESI) were measured on an Agilent Technologies 6110 mass spectrometer.

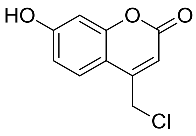

3a

4-(Chloromethyl)-7-hydroxy-2*H*-chromen-2-one (3a): Resorcinol (11.0 g, 100 mmol) was carefully dissolved in H_2SO_4 solution (95%, 90 mL) at 0 °C with stirring. Ethyl 4-chloroacetoacetate (18.1 g, 110 mmol) was slowly added to the solution and the reaction mixture was stirred at 0 °C to room temperature for 5 h. TLC indicated that resorcinol was completely consumed and a fluorescent product was formed. The solution was poured slowly into an ice/water mixture (700 mL) and a large amount of solid was precipitated. The precipitate was filtered and

washed with water several times. It was then dried in an oven to afford a white solid, which is product **3a** (16.3 g, 77.4 mmol, 77.4% yield); ESI-MS *m*/*z*: 211 $[M + H]^+$; ^{1}H NMR (500 MHz, DMSO-d_6) δ 4.94 (s, 2H), 6.41 (s, 1H), 6.75 (d, J = 2.5 Hz, 1H), 6.84 (dd, J_1 = 2.5 Hz, J_2 = 8.8 Hz, 1H), 7.67 (d, J = 8.8 Hz, 1H), 10.64 (s, 1H); ^{13}C NMR (125 MHz, DMSO-d_6) δ 41.36, 102.50, 109.32, 111.02, 113.06, 126.50, 150.95, 155.27, 160.13, 161.46.

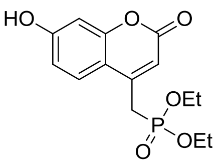

4a

Diethyl ((7-hydroxy-2-oxo-2*H*-chromen-4-yl)methyl) phosphonate (4a): Compound **3a** (860 mg, 4.10 mmol) was suspended in triethyl phosphite (2.72 g, 16.38 mmol), a catalytic amount of potassium iodide was added and the mixture was heated under reflux at 155 °C under N_2 for 4 h. TLC indicated that compound **3a** was completed consumed. The reaction mixture was concentrated using an oil pump to afford compound **4a** as orange-colored oil, which was used in the following reaction without purification. ESI-MS *m*/*z*: 313 $[M + H]^+$.

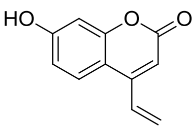

5a

7-Hydroxy-4-vinyl-2*H*-chromen-2-one (5a): Compound **4a** (4.10 mmol) from the last step was dissolved in anhydrous THF (40 mL) in an ice bath. NaH (60%, 0.82 g, 20.5 mmol)

was added to the solution batchwise and it was then stirred at 0 °C for 2 h. Formaldehyde solution (40%, 3 g, 40 mmol) was added dropwise and the reaction mixture was then stirred at rt for 5 h. TLC indicated the formation of three new spots with fluorescence. The solution was evaporated to dryness and then partitioned between ether and brine (v:v, 1:1, 100 mL total). The aqueous layer was further extracted with ether (2 × 50 mL). TLC indicated that the fluorescent products were in the organic layers. The organic layers were combined and condensed and then purified by flash chromatography (petroleum ether:ethyl acetate, 3:1) to afford compound **5a** as a yellow solid (210 mg, 1.12 mmol, 27.3% yield over the last two steps). ESI-MS *m/z*: 189 [M + H]$^+$, 211 [M + Na]$^+$; ^{1}H NMR (500 MHz, DMSO-d_6) δ 5.74 (dd, J_1 = 1.0 Hz, J_2 = 11.3 Hz, 1H), 6.21 (dd, J_1 = 1.0 Hz, J_2 = 17.1 Hz, 1H), 6.35 (s, 1H), 6.74 (d, J = 2.5 Hz, 1H), 6.81 (dd, J_1 = 2.5 Hz, J_2 = 8.8 Hz, 1H), 7.15 (dd, J_1 = 11.3 Hz, J_2 = 17.1 Hz, 1H), 7.74 (d, J = 8.8 Hz, 1H); ^{13}C NMR (125 MHz, DMSO-d_6) δ 102.57, 106.20, 110.36, 111.02, 113.02, 123.96, 126.45, 130.19, 150.65, 155.32, 161.20.

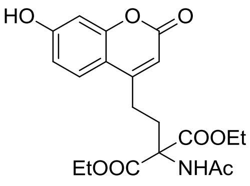

6a

Diethyl 2-acetamido-2-(2-(7-hydroxy-2-oxo-2*H*-chromen-4-yl)ethyl)malonate (6a): Potassium *tert*-butoxide (89.6 mg, 0.800 mmol), diethyl

acetamidomalonate (231 mg, 1.10 mmol) and catalytic amount of tetrabutylammonium bromide (TBABr) were dissolved in anhydrous THF (30 mL) and stirred at 0 °C for 1 h. Compound **5a** (50 mg, 0.27 mmol) was added to the solution and the reaction mixture was heated under reflux at 55 °C overnight. The reaction mixture was concentrated to dryness and partitioned between ethyl acetate and water. TLC indicated that the fluorescent product was in the organic layer. The organic layer was condensed and purified by flash chromatography ($CHCl_3$:CH_3OH, 20:1) to afford a light yellow solid product **6a** (80 mg, 0.20 mmol, 73.1% yield). ESI-MS *m*/*z*: 406 $[M + H]^+$, 428 $[M + Na]^+$; ^1H NMR (500 MHz，DMSO-d_6) δ 1.13 (t, J = 7.1 Hz, 6H), 1.97 (s, 3H), 2.43 (m, 2H), 2.61 (m, 2H), 4.13 (q, J = 7.1 Hz, 4H), 6.09 (s, 1H), 6.72 (d, J = 2.4 Hz, 1H), 6.81 (dd, J_1 = 2.4 Hz, J_2 = 8.8 Hz, 1H), 7.46 (d, J = 8.8 Hz, 1H), 8.46 (s, 1H), 10.56 (s,1H dis. with D_2O); ^{13}C NMR (125 MHz, DMSO-d_6) δ 13.74, 22.15, 25.79, 31.64, 61.83, 65.65, 102.54, 110.04, 110.77, 112.97, 125.80, 155.17, 155.45, 160.15, 161.14, 167.20, 169.40.

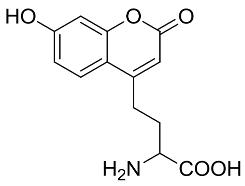

1a

***rac*-(2-(7-Hydroxycoumarin-4-yl)ethyl)glycine (1a):** Compound **6a** (80 mg, 0.20 mmol) was dissolved in HCl (12 M, 30 mL) and heated under reflux at 110 °C in a sealed tube for 4 h. The reaction mixture was evaporated

to dryness and partitioned between ethyl acetate and water (v:v, 1:1, 100 mL total). The aqueous layer was extracted by ethyl acetate (2 × 50 mL). The acid aqueous phase was then lyophilized to give colorless, crystalline product **1a** as a salt of HCl (57 mg, 0.19 mmol, 95% yield). ESI-MS *m*/*z*: 264 $[M + H]^+$, 286 $[M + Na]^+$; ^1H NMR (500 MHz, DMSO-d_6) δ 2.21-2.07 (m, 2H), 3.0-2.85 (m, 2H), 4.02 (s, 1H), 6.13 (s, 1H,), 6.77 (d, J = 2.5 Hz, 1H), 6.84 (dd, J_1 = 2.5 Hz, J_2 = 8.8 Hz, 1H), 7.67 (d, J = 8.8 Hz, 1H), 8.64 (s, 3H), 10.75 (s, 1H dis. with D_2O); ^{13}C NMR (125 MHz, DMSO-d_6) δ: 26.87, 28.65, 51.58, 102.54, 109.58, 110.83, 113.08, 126.25, 155.13, 155.26, 160.34, 161.41, 170.63.

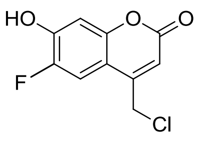

3b

4-(Chloromethyl)-6-fluoro-7-hydroxy-2*H*-chromen-2-one (3b): To a solution 4-fluoro-1,3-dihydroxybenzene (2.89 g, 22.6 mmol) in ice-cold concentrated sulfuric acid (98%, 10 mL) was added ethyl 4-chloroacetoacetate (3.72 g, 22.6 mmol) dropwise. The reaction mixture was then allowed to react at 0 °C to room temperature for 5 h. TLC indicated the disappearance of 4-fluoro-1,3-dihydroxybenzene and the formation of a new fluorescent compound. The reaction mixture was poured into ice-water (100 mL), and large amount of maroon solid was formed immediately. The mixture was filtered, washed with water several times and dried to afford solid product (4.00 g, 78.0% yield). Compound **3b** ($C_{10}H_6ClFO_3$)

ESI-MS *m*/*z*: 229 $[M + H]^+$, 251 $[M + Na]^+$; ^{1}H NMR (500 MHz, DMSO-d_6) δ 4.95 (s, 2H), 6.49 (s, 1H), 6.95 (d, *J* = 7.69 Hz, 1H), 7.66 (d, *J* = 12.13 Hz, 1H).

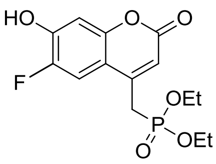

4b

Diethyl (6-fluoro-7-hydroxy-2-oxo-2*H*-chromen-4-yl) methylphosphonate (4b): Compound **3b** (3.00 g, 13.2 mmol) and a catalytic amount of potassium iodide were dissolved in triethyl phosphite (12.79 g, 77.0 mmol) to form a brown solution. It was then heated under reflux at 155 °C for 4 h under N_2. The reaction mixture was concentrated using an oil pump to afford a brown solid. It was resuspended in ethyl acetate and the mixture was put in a freezer set at −20 °C. A solid product was precipated overnight, which was then filtered, washed with ethyl acetate several times and dried to afford brown solid product **4b** (2.80 g, 64.2% yield). The product was used in the following reaction without purification.

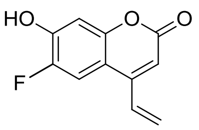

5b

6-Fluoro-7-hydroxy-4-vinyl-2*H*-chromen-2-one (5b): NaH (60%, 256 mg, 6.4 mmol) was added slowly to a solution of compound **4b** (422 mg, 1.28 mmol) in anhydrous THF (15 mL) in an ice bath. The reaction mixture was then stirred at 0 °C for 0.5 h. After an aqueous solution of formaldehyde (40%, 0.96 g, 12.8 mmol) was added

dropwise, the reaction mixture was stirred at room temperature for 5 h. After concentration at reduced pressure, the mixture was partitioned between ether and brine (v/v, 1/1). The ethereal layer was evaporated to dryness to afford a light brown solid (**5b**) (220 mg, 83.0% yield). Compound **5b** ($C_{11}H_7FO_3$) ESI-MS *m*/*z*: 207 $[M + H]^+$, 229 $[M+Na]^+$; ^1H NMR (500 MHz, DMSO-d_6) δ 5.75 (dd, J_1 = 0.8 Hz, J_2 = 11.3 Hz, 1H), 6.19 (dd, J_1 = 0.8 Hz, J_2 = 17.2 Hz, 1H), 6.47 (s, 1H), 6.93 (d, *J* = 7.2 Hz, 1H), 7.15 (dd, J_1 = 11.3 Hz, J_2 = 17.2 Hz, 1H), 7.77 (d, *J* = 11.8 Hz, 1H).

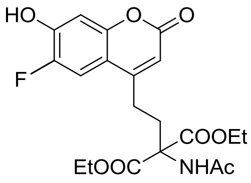

6b

Diethyl 2-acetamido-2-(2-(6-fluoro-7-hydroxy-2-oxo-2*H*-chromen-4-yl)ethyl)malonate (6b): Potassium *tert*-butoxide (673 mg, 6.00 mmol), diethyl acetamidomalonate (652 mg, 3.00 mmol) and a catalytic amount of tetrabutylammonium bromide (TBABr) were dissolved in anhydrous THF (30 mL) and stirred at 0 °C for 1 h. Compound **5b** (206 mg, 1.00 mmol) was added to the solution and the reaction mixture was heated under reflux at 55 °C for 4 h. The reaction mixture was concentrated to dryness and partitioned between ethyl acetate and water. TLC indicated that the fluorescent product was in the organic layer. The organic layer was condensed and the residue was purified by flash chromatography ($CHCl_3$:CH_3OH, 20:1) to afford a

light yellow solid product **6b** (364 mg, 86.0% yield). Compound **4b** ($C_{20}H_{22}FNO_8$) ESI-MS *m*/*z*: 424 [M + H]$^+$, 446 [M + Na]$^+$.

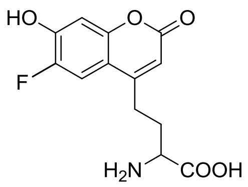

1b

2-Amino-4-(6-fluoro-7-hydroxy-2-oxo-2*H*-chromen-4-yl) butanoic acid (1b): Compound **6b** (80 mg, 0.19 mmol) was dissolved in HCl (30 mL, 12 M) and heated udner reflux at 110 °C for 4 h in a sealed tube. The reaction mixture was concentrated under reduced pressure and precipitated with ethyl acetate. The precipitate was filtered and washed with ethyl acetate several times to afford product **1b** as a HCl salt (50 mg, 83% yield). Compound **1b** ($C_{13}H_{12}FNO_5$) ESI-MS *m*/*z*: 282 [M + H]$^+$, 304 [M + Na]$^+$; ^{1}H NMR (500 MHz, DMSO-d_6) δ 2.15-2.06 (m, 2H), 2.91-2.82 (m, 2H), 4.06 (m, 1H), 6.21 (s, 1H), 6.95 (d, *J* = 7.3 Hz, 1H), 7.63 (d, *J* = 11.7 Hz, 1H), 8.44 (s, 3H); ^{13}C NMR (125 MHz, DMSO-d_6) δ 26.87, 28.65, 51.58, 102.54, 109.58, 110.83, 113.08, 126.25, 155.13, 155.26, 160.34, 161.41, 170.63; ^{19}F NMR (470 MHz, DMSO-d_6) δ −143.44.

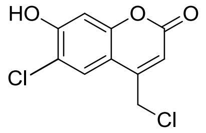

3c

6-Chloro-4-(chloromethyl)-7-hydroxy-2*H*-chromen-2-one (3c): To a solution of 4-chloro-1,3-dihydroxybenzene (14.4 g, 100 mmol) in ice-cold concentrated sulfuric acid (98%, 30 mL)

was added ethyl 4-chloroacetoacetate (16.4 g, 100 mmol) dropwise. The reaction mixture was then allowed to react at 0 °C to room temperature for 5 h. TLC indicated the disappearance of 4-fluoro-1,3-dihydroxybenzene and the formation of a new fluorescent compound. The reaction mixture was poured into ice-water (100 mL), and a large amount of yellow solid was formed immediately. The mixture was filtered, washed with water several times and dried to afford a solid product (17.1 g, 70.0% yield). Compound **3c** ($C_{10}H_6Cl_2O_3$) ESI-MS *m*/*z*: 246 $[M + H]^+$, 268 $[M + Na]^+$; ^1H NMR (500 MHz，DMSO-d_6) δ 4.99 (s, 2H), 6.48 (s, 1H), 6.94 (s, 1H), 7.86 (s, 1H).

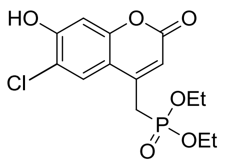

4c

Diethyl (6-chloro-7-hydroxy-2-oxo-2*H*-chromen-4-yl) methylphosphonate (4c): Compound **3c** (2.83 g, 11.6 mmol) and a catalytic amount of potassium iodide were dissolved in triethyl phosphite (7.64 g, 46.0 mmol) to form a brown solution. It was then heated under reflux at 155 °C for 4 h under N_2. The reaction mixture was concentrated using an oil pump to afford a brown solid. It was resuspended in ethyl acetate and the mixture was put in a freezer set at −20 °C. The solid product was precipated overnight, which was filtered, washed with ethyl acetate several times and dried to afford brown solid product **4c** (2.48 g, 62.0% yield). The product was used in the following reaction without further purification.

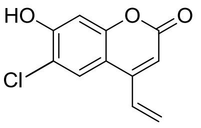

5c

6-Chloro-7-hydroxy-4-vinyl-2*H*-chromen-2-one (5c): NaH (60%, 0.20 g, 5 mmol) was added slowly to a solution of compound **4c** (240 mg, 0.69 mmol) in anhydrous THF (10 mL) in an ice bath. The reaction mixture was then stirred at 0 °C for 0.5 h. After an aqueous solution of formaldehyde (40%, 0.52 g, 6.9 mmol) was added dropwise, the reaction mixture was stirred at room temperature for 5 h. After concentration at reduced pressure, the mixture was partioned between ether and brine (v/v, 1:1). The ethereal layer was evaporated to dryness to afford a light brown solid, which was purified by flash chromatography (petroleum ether:ethyl acetate, 3:1) to afford product **5c** (137 mg, 89.2% yield). Compound **5c** ($C_{11}H_7ClO_3$) ESI-MS *m*/*z*: 223 $[M + H]^+$; ^{1}H NMR (500 MHz，DMSO-d_6) δ 5.74 (d, J = 11.1 Hz, 1H)，6.17 (d, J = 17.1 Hz, 1H), 6.43 (s, 1H), 6.91 (s, 1H), 7.20 (dd, J_1 = 11.1 Hz, J_2 = 17.1 Hz, 1H), 7.92 (s, 1H).

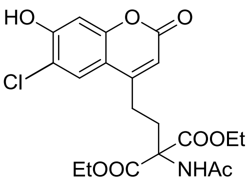

6c

Diethyl 2-acetamido-2-(2-(6-chloro-7-hydroxy-2-oxo-2*H*-chromen-4-yl)ethyl)malonate (6c): Potassium *tert*-butoxide (189 mg, 1.68 mmol), diethyl acetamidomalonate (373 mg, 1.70 mmol) and a catalytic amount of tetrabutylammonium bromide (TBABr) were dissolved in anhydrous THF (30 mL) and stirred at 0 °C for 1 h. Compound **5c** (125 mg, 0.56 mmol)

was added to the solution and the reaction mixture was heated under reflux at 55 °C for 4 h. The reaction mixture was concentrated to dryness and partitioned between ethyl acetate and water. TLC indicated that the fluorescent product was in the organic layer. The organic layer was condensed and the residue was purified by flash chromatography ($CHCl_3$:CH_3OH, 20:1) to afford a light yellow solid product 6c (212 mg, 86.0% yield). Compound **6c** ($C_{20}H_{22}ClNO_8$) ESI-MS *m*/*z*: 440 $[M + H]^+$, 462 $[M + Na]^+$; ^1H NMR (500 MHz, DMSO-d_6) δ 1.14 (t, 6H), 1.98 (s, 3H), 2.38-2.41 (m, 2H), 2.59-2.63 (m, 2H), 4.12-4.17 (m, 4H), 6.17 (s, 1H,), 6.91 (s, 1H), 7.57(s, 1H), 8.49 (s, 1H).

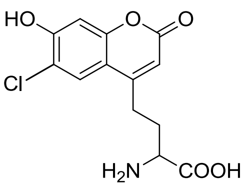

1c

2-Amino-4-(6-chloro-7-hydroxy-2-oxo-2*H*-chromen-4-yl)butanoic acid (1c): Compound **6c** (212 mg, 0.48 mmol) was dissolved in HCl (50 mL, 12 M) and heated under reflux at 110 °C for 6 h in a sealed tube. The reaction mixture was concentrated under reduced pressure and precipitated with ethyl acetate. The precipitate was filtered and washed with ethyl acetate several times to afford product **1c** as a HCl salt (135 mg, 84.3% yield). Compound **1c** ($C_{13}H_{12}ClNO_5$) ESI-MS *m*/*z*: 298 $[M + H]^+$, 320 $[M + Na]^+$; ^1H NMR (500 MHz, DMSO-d_6) δ 2.20-2.03 (m, 2H), 2.97-2.78 (m, 2H), 4.02 (t, J = 6.0 Hz, 1H), 6.18 (s, 1H), 6.95 (s, 1H), 7.79 (s, 1H); ^{13}C NMR (125 MHz, DMSO-d_6) δ 26.62,

28.45, 51.71, 103.71, 110.66, 111.68, 117.16, 125.46, 153.34, 154.31, 156.55, 159.86, 170.71.

Protein expression

To determine whether compound **1a** is incorporated into the protein with high efficiency and fidelity, an amber stop codon was substituted for Ile38 in *E.coli* Thioredoxin-1 (TRX). The plasmid pEVOL-CouRS-D8 was cotransformed with a plasmid carrying the Thioredoxin38TAG gene (pET-TRX38TAG) into *E. coli* BL21-DE3 cells. Bacteria were grown at 37 °C in LB medium for 2.5 h until OD_{600} = 1.0, at which point 2 mM racemic **1a** was added to the culture. The bacteria were grown at 37 °C for 30 min and then protein expression was induced by the addition of 0.02% arabinose and 1 mM isopropyl-β-D-thiogalactopyranoside (IPTG). After another 5 h, cells were harvested by centrifugation. The TAG38 mutant thioredoxin was purified by Ni-NTA affinity chromatography under native conditions. Analysis of the purified protein by SDS-PAGE showed that full-length thioredoxin was expressed only in the presence of **1a** (Figure 2).

A

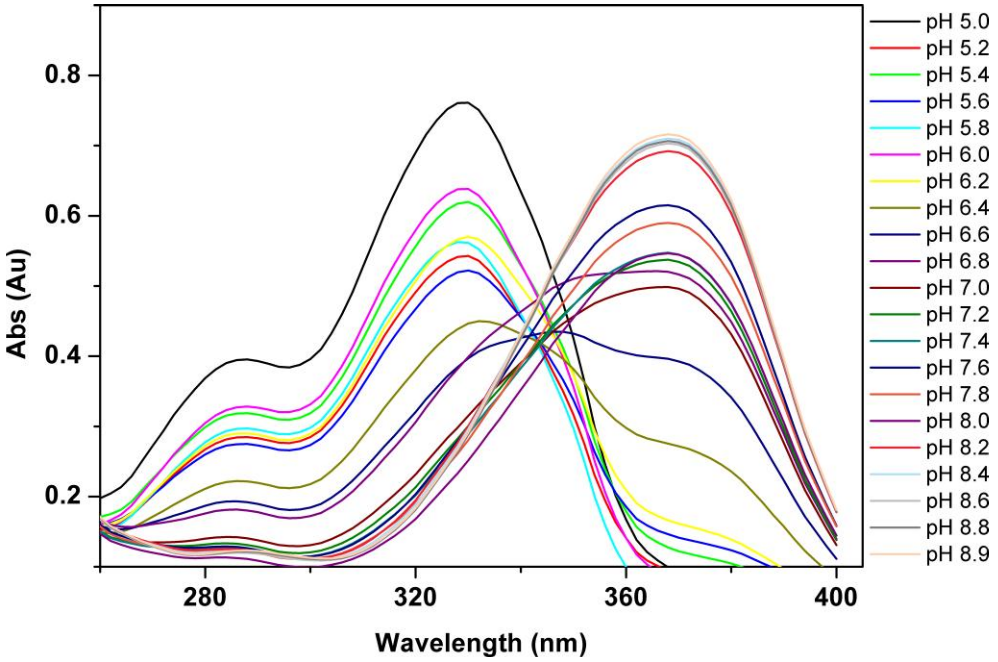

B

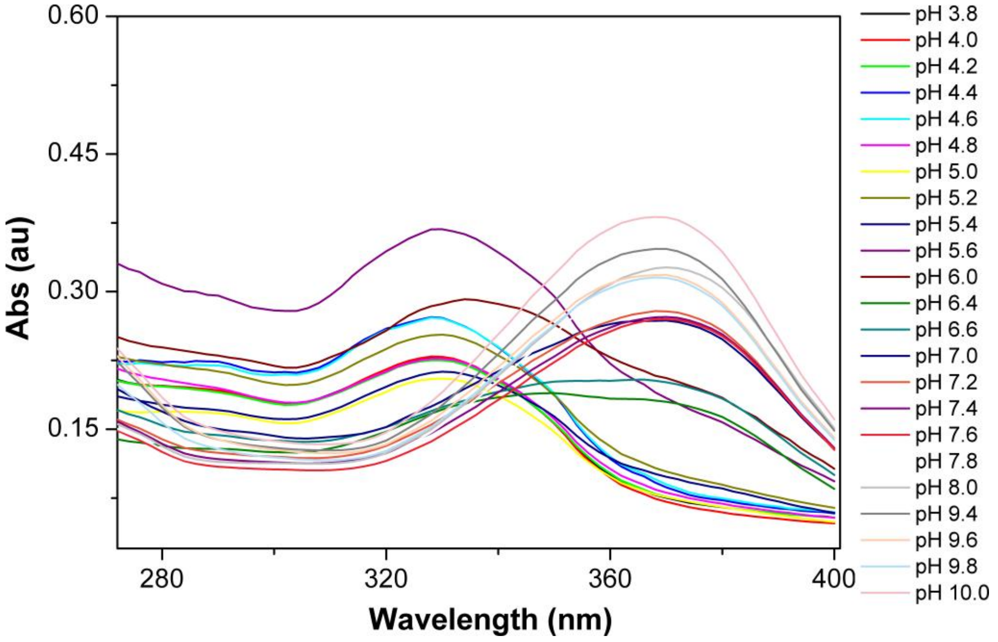

Figure S1: Absorption spectra of **1b** and **1c** at different pH. (A) Absorption spectrum of 50 μM **1b** in 200 mM sodium phosphate buffer (pH 5.8–8.0), 200 mM sodium acetate buffer (pH 3.7–5.6) or 50 mM Tris-HCl Buffer (pH 8.2–8.9). (B) Absorption spectrum of 25 μM **1c** in 200 mM sodium phosphate buffer (pH 5.8–8.0) or sodium acetate buffer (pH 3.7–5.6).
